# Impact of an Educational Film on Parental Knowledge of Children with Cerebral Palsy

**DOI:** 10.1155/2014/573698

**Published:** 2014-03-05

**Authors:** Shilpa Khanna Arora, Anju Aggarwal, Hema Mittal

**Affiliations:** ^1^Department of Pediatrics, Vardhman Mahavir Medical College and Safdarjung Hospital, New Delhi 110029, India; ^2^Department of Pediatrics, University College of Medical Sciences and GTB Hospital, Delhi 110095, India

## Abstract

Parents of children with cerebral palsy (CP) must have knowledge about the disease and its management to improve the outcome. This uncontrolled interventional trial was carried out to evaluate the parental knowledge of CP and assess the impact of an educational programme on it. Preset questionnaires were filled before and 1 week after a single session educational programme using an educational film. Out of a total of 53 subjects, majority (75.5%) were from lower socioeconomic status. Initially, none knew the correct name of child's illness; afterwards 45.3% could name it. When compared to previous status, there occurred significant improvement in the knowledge of parents after viewing the film with regard to knowing the cause of CP, knowing that motor involvement was predominant in CP, knowledge regarding curability of the disease, and knowledge about special schooling (*P* < 0.05). Change in knowledge was not related to socioeconomic or educational status (*P* > 0.05). Majority (94.3%) found the film useful and 96.2% learned how they could help in the management of their children. Parental knowledge of CP is inadequate which can be improved by incorporating such educational programmes in special clinics to improve management.

## 1. Introduction

Cerebral palsy (CP) is the most common physical disability in childhood [1]. Incidence of CP has been estimated to be 2–2.8 per 1000 live births [2]. CP cannot be cured but early intervention therapy can help achieve functional abilities that facilitate independence and improve quality of life [3]. It is known that a supportive home environment is one of the factors that can favourably determine the outcome of CP in a child. Parental involvement is vital in the process of rehabilitation and care of such children [4]. Thus parents of children with cerebral palsy must have knowledge about the disease and its management. This would help in planning therapy to achieve functional abilities and improve quality of life. At present there are very few studies available regarding parental knowledge on cerebral palsy. Review of the current literature shows that majority of the parents of children with CP lack the basic knowledge regarding the disease, its causation, prognosis, treatment modalities, and the outcome [3, 5–9]. Also there is paucity of studies carrying out any intervention and evaluating the response of that intervention on the parental knowledge regarding CP. Thus this study was carried out to determine the present knowledge of parents of their child's cerebral palsy and to evaluate the impact of a single session educational programme on their knowledge.

## 2. Material and Methods

This uncontrolled interventional trial was carried out in a tertiary care hospital from August 2010 to March 2011. Approval was obtained from the institutional ethical committee. A written informed consent was obtained from the parents. Subjects were parents (either mother or father or both) of recently diagnosed (≤1 month) cases of CP presenting to the clinic. Guardians/caretakers other than biological parents and the parents who had already watched the educational film were excluded from the study. The parental knowledge regarding CP was assessed by filling a questionnaire by the investigator in the language they understood. It included questions about the name of the disease, its probable aetiology, treatment options, and rehabilitation of the child.

Following that, the subjects were shown an educational film and asked to follow up after one week. The film which is in Hindi language talks about the aetiology, management, and the role of parents in managing CP. It also describes briefly the eight spheres of development and how they are involved in CP as language, play, social communication, and so forth, as well as the fact that motor involvement was predominant in cerebral palsy. It emphasized the fact that a child with cerebral palsy can be rehabilitated to do activities of daily living and some of them are mentally normal. No two children of cerebral palsy are the same. Film explained the role of parental involvement in training of the child. The same questionnaire was filled up by the investigator again with a few additional questions based on the film on the follow-up visit.

The impact of the educational film on the parental knowledge of CP was assessed by comparing the responses before and after watching the educational film. Knowledge about various aspects of cerebral palsy was classified as correct or incorrect/know or do not know. Knowledge about the various aspects of the film was assessed as percentages. Statistical analysis was done using SPSS Version 14.0, SPSS Inc., Chicago. The change in parental responses to the questionnaire before and after showing the film was analysed using the McNemar test. *P* value of <0.05 was taken as significant. Chi-square test/Fisher exact test was used to compare the response to individual questions with each of the demographic parameters.

## 3. Results

Parents of a total of 53 children (35 males and 18 females) who were recently diagnosed with cerebral palsy were included. The age of the children ranged from 6 to 72 months. Majority (30/53 or 56.6%) were of first birth order. There was history of significant perinatal events in 45/53 (84.9%) children. The type of CP from which the children suffered was spastic quadriplegic (71.7%), diplegic (18.9%), hemiplegic (5.7%), and dyskinetic (3.8%). Associated comorbidities in children were drooling (66.0%), behavioural problems (64.2%), seizures (60.4%), visual disability (32.1%), contractures (17.0%), and hearing disability (11.3%). The age at which parents noted symptoms in their children ranged from 1 to 36 months (mean ± SD = 10.02 ± 6.72) whereas age at which diagnosis of CP was established ranged from 2 to 72 months (mean ± SD = 19.64 ± 15.40).

Majority (77.4%) of the parents belonged to upper-lower or lower socioeconomic strata according to modified Kuppuswamy scale [10]. The age of the fathers ranged from 23 to 64 years (mean ± SD = 31.02 ± 7.42) and that of mothers ranged from 20 to 55 years (mean ± SD = 27.11 ± 6.46). The educational status of fathers was illiterate (15.1%), primary (9.4%), intermediate (58.5%), and graduate (17%). The educational status of mothers was illiterate (37.7%), primary (9.4%), intermediate (45.3%), and graduate (7.5%). The interviewees were mothers in majority of the cases (31/53 or 58.5%) whereas fathers constituted 15.1% (8/53) and both parents were interviewed in 26.4% (14/53) of cases.

The parents' responses to the questions before and after viewing the film have been tabulated in Table 1. In the first interview 60.4% of parents answered correctly when asked about their knowledge regarding the aspect of development involved in CP by saying that the child had difficulty in holding neck/sitting/standing or walking. This figure rose significantly to 79.2% after watching the film. Thirteen (24.5%) of the parents were not aware of any of the treatment modalities of CP prior to watching of the film. After the film almost all knew about at least one treatment modality. The most common treatment modality which the parents knew of was physiotherapy.

The responses to a set of additional questions related to the film and its content which were asked in the follow-up interview have been tabulated in Table 2. The film described that child development is divided into 8 parts; 64% could recall that correctly and 69.8% remembered that the main part involved in CP was motor development. Majority (96.2%) could tell how they can contribute to better rehabilitation of their child.

There was no significant relation between the sex of the child; socioeconomic status; previous treatment or follow up at some other institutions; perinatal events; type of CP; or educational status of the parents on the change in parental knowledge of CP as assessed by chi-square/Fisher's exact test (*P* > 0.05).

## 4. Discussion

The majority of the subjects in the present study were unaware of basic knowledge of the disease like the correct name of the illness, its causation, the aspect of development involved, progression/course, curability, treatment modalities, and possibility of schooling. Thus there is lack of parental knowledge regarding cerebral palsy which is in accordance with previous studies [3, 5–9].

It has been seen that the parents of patients suffering from CP receive very little information from the treating physicians, nurses, and therapists and have many queries about the disease which tend to remain unanswered [8, 9]. This leads to lack of confidence interfering with the process of decision making [5, 7]. Also there is lack of educational activities to improve the parental knowledge; hence it leads to poor compliance with the treatment and interferes with the process of rehabilitation. There is need to better inform and educate family about the diagnosis, treatment, and prognosis so that the parents can make better decisions about their children and alleviate the stress that arises from ignorance and uncertainty [5, 8]. The best method of rehabilitative training of the child is to make the training activities part of the child's daily routine activities which can be accomplished only with parental involvement [4]. Greater understanding would form a relationship of trust between the families and the health professionals resulting in provision of better care and hence an improved outcome. In a study evaluating the knowledge of parents about child development in normal children, it was observed that the parents who have better knowledge about the stages of child's development can take better care of their children and this positively affects the child's development [11]. The same would be true for parents of disabled children as well.

There is paucity of studies carrying out any intervention like showing an educational film to improve the parental knowledge. In this study there occurred significant improvement in parental knowledge of CP after watching the educational film. Karande et al. carried out a single session structured educational programme comprising flash cards which also significantly improved the parental knowledge of CP [3]. In our study parents' knowledge about the disease, its probable aetiology and the fact that disease is not totally curable increased significantly. This is likely to have significant impact on parents' behaviour and help in management of the child.

The study population comprised almost double the number of male children as compared to female children which is greater than the usually observed sex ratio of 1.4 : 1 [12]. This disparity could be due to the preferential health seeking behaviour of parents for the male child. The mean age around which parents noticed their child's symptoms was 10 months, whereas mean age at diagnosis was around 20 months. This gap could probably be due to the fact that majority of the children were first in birth order hence parents being unaware of normal developmental milestones, due to delay in referral or establishment of definitive diagnosis of CP by doctors. It was observed that the majority of the interviewees were mothers. This is in accordance with previous literature which shows that more mothers than fathers are the primary care takers for their disabled children [3, 13, 14].

This study has the limitation of a brief period of follow-up, that is, 1 week, to assess the impact of the educational programme on the parental knowledge. There is need to carry out further studies to assess the long term impact of such an educational programme preferably as repeated sessions on the parental knowledge. The intervention that is the educational film used in this study was devised for Indian, Hindi speaking subjects; hence the results of the impact of this film cannot be applied to the other populations. There is need to develop similar educational films in local languages in accordance with the regional cultures and ethos in order to facilitate the understanding and improve the therapeutic outcome in CP children in other regions of the world.

As there was no significant correlation between socioeconomic and educational status of parents to the change in parental knowledge of CP hence such an educational film can help in improving parental knowledge of CP irrespective of parents' educational or socioeconomic background.

Thus we conclude that parental knowledge of CP is lacking and an intervention such as an educational film will have a positive impact on the parents' knowledge; hence measures such as educational film viewing should be a part of the management of cerebral palsy in special clinics.

## Figures and Tables

**Table 1:** Parents response before and after viewing the educational film (*n* = 53).

Question	Response	Before	After	*P* value
				McNemar
What is the name of the illness from which your child suffers?	Correct	0 (0%)	24 (45.3%)	<0.001
	Incorrect	53 (100%)	29 (54.7%)	
What do you think is the cause of this disorder “cerebral palsy”?	Correct	14 (26.4%)	48 (90.6%)	<0.001
	Incorrect	39 (74.5%)	5 (9.4%)	
What aspect of child development is involved in cerebral palsy?	Correct	32 (60.4%)	42 (79.2%)	<0.001
	Incorrect	21 (39.6%)	11 (20.8%)	
Do you think that this disease will increase in severity?	Correct	19 (35.8%)	53 (100%)	<0.001
	Incorrect	34 (64.2%)	0 (0%)	
Will this disease be totally cured?	Correct	21 (39.6%)	50 (94.3%)	<0.001
	Incorrect	32 (60.4%)	3 (5.7%)	
Do you think that such children can get schooling or not?	Correct	33 (62.3%)	52 (98.1%)	<0.001
	Incorrect	20 (37.8%)	1 (1.9%)	
Are you aware of special schools for such children?	Yes	12 (22.6%)	35 (66.0%)	<0.001
	No	41 (77.4%)	18 (34.0%)	
Do you think that this disorder is preventable?	Correct	13 (24.5%)	48 (90.6%)	<0.001
	Incorrect	40 (75.5%)	5 (9.4%)	
How can CP be prevented?	Correct	11 (20.8%)	44 (83.0%)	<0.001
	Incorrect	42 (79.2%)	9 (17.0%)	

**Table 2:** Parents responses regarding the film and its content (*n* = 53).

Question	Yes/Correct	No/Incorrect
Was the film useful?	50 (94.3%)	3 (5.7%)
Did you acquire any additional knowledge about the disease?	46 (86.8%)	7 (13.2%)
Did you acquire any additional knowledge about the treatment?	44 (83.0%)	9 (17.0%)
Did you acquire any additional knowledge about the trainability of the child?	51 (96.2%)	2 (3.8%)
In the film child's development was divided into how many parts?	34 (64.2%)	19 (35.8%)
What are the main parts involved in cerebral palsy?	37 (69.8%)	16 (30.2%)
How can you contribute to better training of your child?	51 (96.2%)	2 (3.8%)
